# Meiotic H3K9me2 distribution is influenced by the ALG-3 and ALG-4 pathway and by poly(U) polymerase activity

**DOI:** 10.17912/micropub.biology.000455

**Published:** 2021-09-14

**Authors:** Yini Li, Matthew Snyder, Eleanor M. Maine

**Affiliations:** 1 Department of Biology, Syracuse University, Syracuse, NY

## Abstract

Histone modifications influence gene expression and chromosome dynamics by altering chromatin structure and recruitment of nonhistone proteins. Dimethylation of histone H3 on lysine 9 (H3K9me2) is a conserved modification often found within heterochromatin. During first meiotic prophase when homologous chromosomes undergo pairing and synapsis, immunolabeling of *C. elegans *male germ cells detects a relatively high H3K9me2 level on the single X chromosome and a relatively low H3K9me2 level on synapsed autosomes. This H3K9me2 distribution is influenced by several components of the small RNA machinery, including: EGO-1 RNA-directed RNA polymerase (RdRP); DRH-3 helicase; EKL-1, a Tudor domain protein; CSR-1 Argonaute; and RRF-3 RdRP. EGO-1, DRH-3, and EKL-1 function together to generate/stabilize 22G RNAs in the germ line. A subset of these 22G RNAs function together with CSR-1 to ensure correct gene expression. RRF-3 RdRP functions in biogenesis of 26G RNAs that feed into two germline regulatory mechanisms mediated by ERGO-1 Argonaute and the redundant ALG-3 and ALG-4 Argonaute proteins. Here, we report that meiotic H3K9me2 distribution is influenced by ALG-3 and ALG-4, as well as by two other factors required for 26G RNA synthesis, ERI-1 and ERI-5. Moreover, meiotic H3K9me2 distribution is influenced by activity of the poly(U) polymerases, PUP-1 (aka CDE-1, CID-1) and PUP-2.

**Figure 1. Disruptions in the ALG-3 and ALG-4 pathway or PUP activity alter meiotic H3K9me2 distribution:**
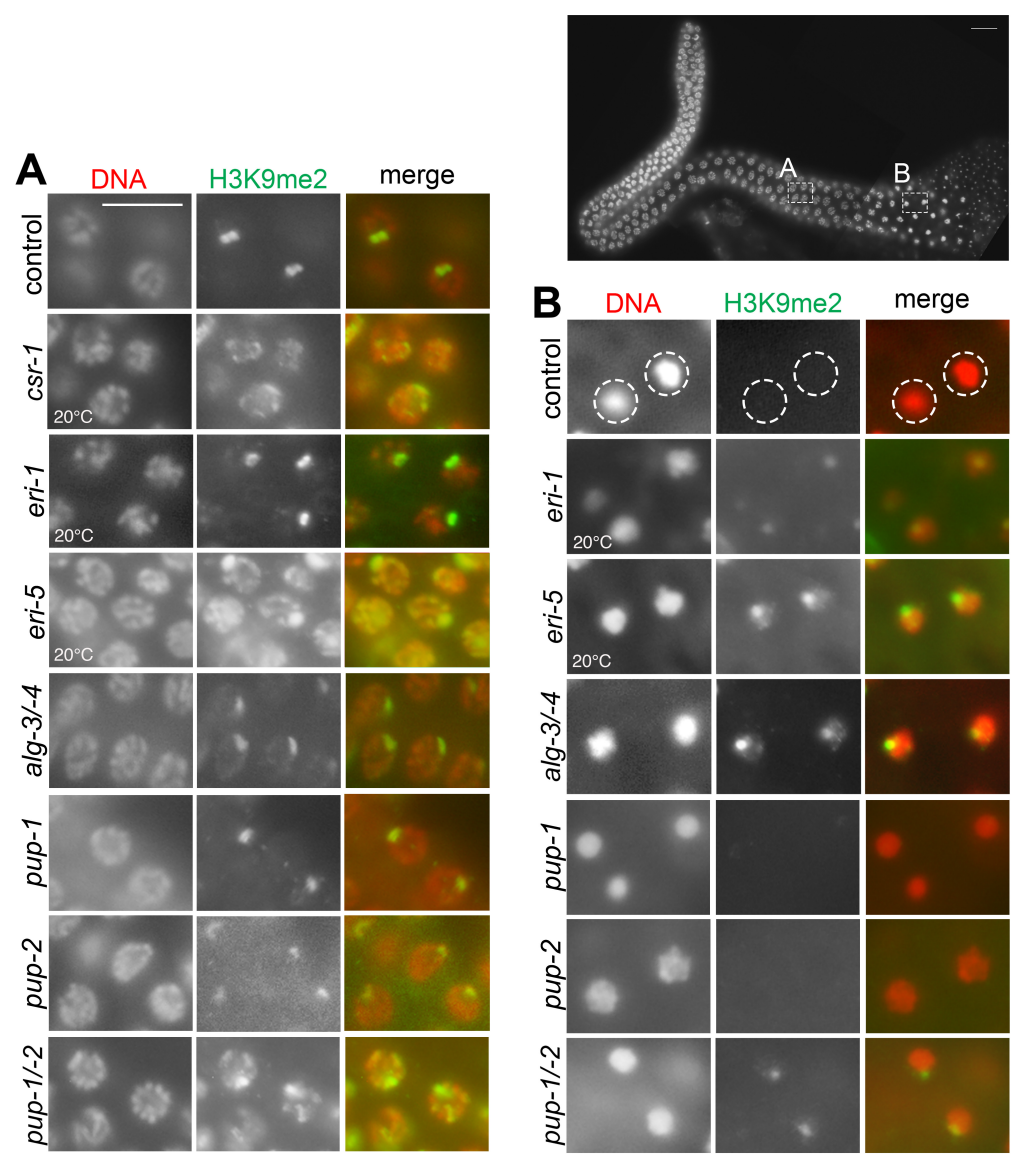
Upper right image shows a dissected adult male gonad labeled with the DNA dye, DAPI, to visualize nuclei. Representative images show (A) pachytene nuclei and (B) karyosomes in adult male germ lines that have been immunolabeled with anti-H3K9me2 antibody. Animals were cultured at 25°C except where noted. (A) Controland *csr-1(om135)* germ line images illustrate the wildtype and ectopic H3K9me2 labeling patterns described in the text. The H3K9me2 signals in *eri-1(mg366)* single mutant, *alg-4(ok1041);alg-3(tm1155)* double mutant (for readability, labeled *alg-3/-4* in the figure), *pup-1* single mutant, and *pup-2* single mutant males resemble the control strain. In each case, one region of relatively strong signal is visible compared to weaker signal across the rest of the chromosomes. In contrast, multiple small H3K9me2 foci are visible in *eri-5(tm2528)* single mutant and *pup-1/-2(om129)* M+Z- double mutant males. (B) Karyosome nuclei lack H3K9me2 signal in control males and in *pup-1(tm1021)* single mutant and *pup-2(tm4344)* singlemutant males. In contrast, karyosomes retain a focus of H3K9me2 signal in *eri-1(mg366)* single mutant, *eri-5(tm2528)* single mutant, *alg-4(ok1041);alg-3(tm1155)* double mutant, and *pup-1/-2(om129)* M+Z- double mutant males. We note that control animals have the same H3K9me2 labeling pattern when grown at 20°C and 25°C. Here, the control strain and several mutant strains carry *him-8(e1489)* to facilitate production of males. HIM-8 protein is critical for X chromosome pairing and, in its absence, hermaphrodites produce frequent nullo-X gametes that result in XO male offspring. Other strains were maintained as male/hermaphrodite populations by mating. See Methods. Scale bar = 16 um.

## Description

Histone modifications are precisely controlled within the developing germ line. During first meiotic prophase in *C. elegans*, H3K9me2 immunolabeling detects a relatively bright focus of signal on unsynapsed chromosomes and a relatively weak, diffuse signal on synapsed chromosomes (Bean *et al.* 2004, Kelly *et al.* 2002, Reuben and Lin 2002). The naturally occurring unsynapsed chromosome is the single male X. Examples of other chromatin that is enriched for H3K9me2 at pachytene stage include single-copy free chromosomal duplications, chromosomes that fail to pair due to certain mutations, and multicopy extrachromosomal arrays. In all cases, the enriched H3K9me2 signal decreases sharply as nuclei move into diplotene stage, and H3K9me2 is not detected in diakinesis nuclei. As the naturally occurring example of H3K9me2 enrichment, the male X is commonly used to evaluate this meiotic targeting phenomenon (Fig. 1). We previously showed that meiotic H3K9me2 distribution is altered in males lacking CSR-1 Argonaute or factors required for accumulation of CSR-1-associated 22G RNAs, including EGO-1 RdRP, DRH-3 helicase,and the Tudor domain protein, EKL-1 (Maine *et al*. 2005, She *et al.* 2009). In mutants lacking any of these factors, pachytene H3K9me2 signal intensity is reduced on unsynapsed chromosomes and, in all cases except *ego-1(null)* mutants, ectopic H3K9me2 foci are detected on synapsed chromosomes. Interestingly, as in wildtype, H3K9me2 signal is not detected in diakinesis nuclei in these mutants. CSR-1 pathway activity is essential for germ line development where it is thought to license the correct pattern of gene expression (Yigit *et al.* 2006, Ashe *et al.* 2012, Shirayama *et al*. 2012). EGO-1, DRH-3, and EKL-1 are critical for biogenesis and stability of 22G RNAs that associate with CSR-1 (reviewed in Billi *et al.* 2014).

RRF-3 RdRP is essential for biogenesis of 26G RNAs that participate in the ALG-3 and ALG-4 Argonaute pathway during spermatogenesis and the ERGO-1 Argonaute pathway during oogenesis (Billi *et al.* 2014). We previously observed an essentially wildtype H3K9me2 immunolabeling pattern in *rrf-3(pk1426)* males during first meiotic prophase that then persists (presumably on the X chromosome) beyond diplotene and is visible on karyosomes in the late spermatogenesis condensation zone as described by Shakes *et al.* (2009) (Maine *et al.* 2005). We observed wildtype H3K9me2 distribution in *ergo-1(tm1860)* single mutant, *alg-3(tm1155)* single mutant, and *alg-4(ok1041)* singlemutant males (She *et al.* 2009), but we did not test *alg-4(ok1041);alg-3(tm1155)* double mutants at that time. We subsequently hypothesized that the *rrf-3(pk1426)* H3K9me2 phenotype may reflect impaired ALG-3 and ALG-4 pathway activity, since these Argonaute proteins together are critical during spermatogenesis (Han *et al.* 2009, Conine *et al.* 2010, 2013) at the time when the H3K9me2 signal typically decreases.

Here, we follow up on our earlier results to report meiotic H3K9me2 distribution in *eri-1(mg366)* single mutant, *eri-5(tm2528)* single mutant, and *alg-4(ok1041);alg-3(tm1155)* double mutant males. ERI-1, an exoribonuclease, and ERI-5, a Tudor domain protein, are both required for 26G RNA synthesis. We also evaluated H3K9me2 distribution in *pup-1(tm1021)* single mutant, *pup-2(tm4344)* single mutant, and *pup-1/-2(om129)* double mutant males. PUP-1 (aka CDE-1, CID-1) and PUP-2 are members of the poly(U) polymerase family responsible for adding non-templated uridine(s) to the 3’ end of RNA. PUP-1 is implicated in adding 3’ uridine to 22G RNAs that associate with CSR-1 and WAGO-4 Argonautes (van Wolfswinkel *et al.* 2009, Xu *et al.* 2018) and to microRNAs (Vieux *et al.* bioRxiv preprint). PUP-2 promotes germline development redundantly with PUP-1 under conditions of heat stress (Li and Maine 2018) and has been implicated in regulating stability of *let-7* miRNA (Lehrbach *et al.* 2009). U-tailing of small RNAs may influence their stability and/or Argonaute associations, as has been suggested (Billi *et al.* 2014, Xu *et al.* 2018).

We immunolabeled H3K9me2 in adult *him-8* control males raised at 20°C and 25°C and in various mutant backgrounds. We tested *alg-4(ok1041);alg-3(tm1155)* double mutant, *eri-1(mg366)* single mutant, *eri-5(tm2528)* single mutant, *pup-1(tm1021)* single mutant, *pup-2(tm4344)* single mutant, and *pup-1/-2(om129)* double mutant males. The *alg-4(ok1041);alg-3(tm1155)* double mutant, *pup-1(tm1021)* single mutant, *pup-2(tm4344)* single mutant, and *pup-1/-2(om129)* double mutant phenotypes are all temperature sensitive, and these mutants were therefore tested at restrictive temperature (25°C). The *pup* single and double mutants were F1 (M+Z-) offspring (where M indicates maternal genotype and Z indicates embryonic genotype) of heterozygous hermaphrodites (see Methods). We note that *pup-1/-2(om129)* M+Z- males all produce sperm, although many of those sperm have abnormal morphology and are fertilization defective (Li and Maine 2018). H3K9me2 immunolabeling in pachytene nuclei appears wildtype in *eri-1(mg366)* single mutant, *alg-4(ok1041);alg-3(tm1155)* double mutant, *pup-1(tm1021)* single mutant, and *pup-2(tm4344)* single mutant males; in each case, a strong signal is visible in one region of the genome, and weak, diffuse signal is visible elsewhere across the genome (Fig. 1A)*.* In contrast, multiple discrete H3K9me2 foci are visible in pachytene nuclei of *pup-1/-2(om129) M*+Z- double mutant and *eri-5(tm2528)* single mutant males (Fig. 1A). In spermatogenic nuclei, H3K9me2 is not detected in *pup-1* or *pup-2* single mutants, as it is not in the control (Fig. 1B). In contrast, H3K9me2 is detected in karyosomes in *alg-4(ok1041);alg-3(tm1155)* double mutant, *eri-1(mg366)* single mutant, *eri-5(tm2528)* single mutant,and *pup-1/-2(om129)* M+Z- double mutant males (Fig. 1B). We note that H3K9me2 appears wildtype *pup-1/-2(om129)* F1 (M+Z-) doublemutants at 20°C, consistent with the temperature-sensitivity of other aspects of the *pup* phenotype (Spracklin *et al.* 2017, Li and Maine 2018).

Our working model is that the delayed H3K9me2 turnover in *rrf-3(pk1426)* single mutant, *alg-4(ok1041);alg-3(tm1155)* double mutant, *eri-1(mg366)* single mutant, and *eri-5(tm2528)* singlemutant malesreflects a role for the ALG-3 and ALG-4 pathway in chromatin regulation during spermatogenesis. The broader H3K9me2 phenotype in *pup-1/-2(om129)* M+Z- 25°Cmutants may reflect impaired activity of both the CSR-1 pathway and the ALG-3 and ALG-4 pathway, perhaps due to misrouting of siRNAs that would typically associate with these Argonaute proteins. CSR-1 promotes spermatogenesis, and it is implicated as functioning downstream of ALG-3 and ALG-4 to regulate gene expression during spermatogenesis (Conine *et al.* 2010, 2013; Charlesworth *et al.* 2021) although CSR-1 does not appear to act directly downstream of ALG-3 and ALG-4 in all cases (Nguyen and Phillips 2021). Misrouting of siRNAs may occur, at least in part, due to absence of 3’ U-tailing as has been proposed by van Wolfswinkel *et al.*. (2009), Xu *et al.* (2018), and others.

## Methods

**Immunocytochemistry and DAPI staining:** Gonads were dissected from L4 + 24 hr adults and immunolabeled with anti-H3K9me2 antibody as described (Maine *et al.* 2005; She *et al.* 2009). Tissue was fixed in 3% PFA/PBS solution for 5 min. Mouse anti-H3K9me2 (Abcam 1220) primary antibody and Alexa Fluor 488-conjugated goat anti-mouse secondary antibody were each used at a 1:200 dilution. Tissue was stained with DAPI in the penultimate wash. Slides were observed with a Zeiss Axioscope or Leica DM5500 microscope. Images of H3K9me2 immunolabeling in different genetic backgrounds were taken at the same exposure.

## Reagents

**Strains used in this study:**

**Table:** 

**Strain**	**Genotype & Linkage Group**	**Notes**
CB1489	*him-8(e1489)* LG IV	
EL502	*eri-1(mg366) him-8(e1489)* LG IV	
EL567	*alg-4(ok1041);alg-3(tm1155)* LG III;IV	Temperature-sensitive phenotype. Mating population was maintained at 20°C.
EL609	*csr-1(om135) him-8(e1489)/mIs11 him-8(om133)* LGIV	Strain construction is described below.
EL624	*pup-1/-2(om129)/qC1gfp;him-8(e1489)* LG III;IV	*om129* deletes the adjacent *pup-1* and *pup-2* genes. Temperature-sensitive phenotype.
EL625	*pup-2(tm4344);him-8(e1489)* LG III;IV	Temperature-sensitive phenotype.
EL690	*pup-1(tm1021)/qC1gfp;him-8(e1489)* LG III; IV	Temperature-sensitive phenotype.
FX2528	*eri-5(tm2528)* LG IV	Maintained as a mating population.
PD4792	*mIs11[myo-2p::GFP + pes10p::GFP + gut-promoter::GFP]* LG IV	

We used CRISPR-Cas9 editing to generate *csr-1(om135)* in the CB1489 *him-8(e1489)* strain background and to generate *him-8(om133)* in the PD4792 *mIs11* balancer strain background. For each mutation, we injected sets of sgRNAs designed to delete a large portion of the gene. *csr-1(om135)* contains a deletion with breakpoints in exon 1 and 11. The deletion removes 3530 nucleotides corresponding to (1) most of the coding region for the longer CSR-1A isoform and (2) the start codon plus most of the coding region for the shorter CSR-1B isoform. The sterile phenotype is 100% penetrant. *him-8(om133)* contains a deletion with breakpoints in exon 4 and 6, removing the 3’ portion of the coding region. The Him phenotype is 100% penetrant.
